# Effect of *Nelumbo nucifera* Petals Extract on Antioxidant Activity and Sperm Quality in Charolais Cattle Sperm Induced by Mancozeb

**DOI:** 10.3390/plants11050637

**Published:** 2022-02-26

**Authors:** Jiraporn Laoung-on, Churdsak Jaikang, Kanokporn Saenphet, Paiwan Sudwan

**Affiliations:** 1 Department of Anatomy, Faculty of Medicine, Chiang Mai University, Chiang Mai 50200, Thailand; jiraporn_laoung@cmu.ac.th; 2 Graduate School, Doctor of Philosophy Program in Anatomy, Faculty of Medicine, Chiang Mai University, Chiang Mai 50200, Thailand; 3 Toxicology Section, Department of Forensic Medicine, Faculty of Medicine, Chiang Mai University, Chiang Mai 50200, Thailand; churdsak.j@cmu.ac.th; 4 Department of Biology, Faculty of Science, Chiang Mai University, Chiang Mai 50200, Thailand; kanokporn.saenphet@cmu.ac.th

**Keywords:** *Nelumbo nucifera* petal extract, flavonoid, antioxidant, sperm quality, mancozeb

## Abstract

The white *Nelumbo nucifera* petals aqueous extraction (NAE) was prominent in phytochemical content, antioxidant activity, and enhanced rat sperm viability induced by FeSO_4_, a heavy metal. Mancozeb (MZ) contains heavy metals and is widely used for fungal control in agriculture and industry. It induces oxidative stress and causes of spermatogenesis and reproductive organs’ abnormalities in both humans and animals. The aims of the present study were to investigate the effects of white *Nelumbo nucifera* petals aqueous extraction (WNAE) on sperm quality in cattle sperm induced by MZ. Moreover, this study investigated phytochemical compounds by liquid chromatography-mass spectrometry. A protein profile related to sperm quality with SDS-page and sperm energy preservation for each treatment was determined. The results found nine phytochemical compounds, in which quercetin-3-O-arabinoglycoside was a major flavonoid that was found in the WNAE. MZ induced free radicals in cells, leading to LPO and protein oxidation, while decreasing sperm motility, sperm viability, acrosome integrity, and normal sperm morphology. The cattle sperm found four proteins related to sperm quality including MWs of 17, 31, 34, and 55 kDa. The WNAE effectively increased energy preservation, sperm motility, sperm viability, acrosome integrity, and normal sperm morphology. The WNAE enhanced sperm qualities by reducing oxidative stress. It might be suggested that WNAE has benefits for sperm preservation which may be used to guard against toxicity in animals or humans exposed to MZ contaminants.

## 1. Introduction

*Nelumbo nucifera* Gaertn. (*N*. *nucifera*), called “Bualuang” in Thai, is an aquatic perennial flowering plant, of the Nelumbonaceae family, which is known as sacred lotus, Indian lotus, bean of India, and Chinese water lily, and can be found mainly in Asia [1]. Various parts of *N*. *nucifera* have been used in traditional medicine [2]. The red *N*. *nucifera* flower has been reported to be a famous perennial aquatic herb [3] and the red long flower’s stamen was recognized as a traditional drug by the National List of Essential Medicines, Thailand [4]. Due to the high economic demand for stamens, the lotus petals become agricultural waste. The phytochemical study using a high-performance liquid chromatography (HPLC) method found that the petals of *N. nucifera* contained phytochemical components, including quercetin, gallic acid, catechin, and *p*-hydroxybenzoic acid, as well as the white *N*. *nucifera* petals aqueous extraction (NAE) which had enhanced rat sperm viability, more phytochemical and antioxidant activity properties than red NAE [5]. However, there are no reports of white NAE for use against mancozeb (MZ) toxicity.

Male reproductive dysfunction is an early problem in men and causes emotional stress [1]. The sperm abnormality is one cause of male reproductive dysfunction that may have occurred from the reducing capability of macromolecule of the sperm including lipids and proteins [6,7]. Four proteins had MWs with 17, 31, 34, and 55 kDa reported to be associated with the sperm motility, acrosome reaction, sperm fertilization, and sperm viability, respectively [8,9,10]. Medicinal plants are often used to enhance male reproductive efficiency, such as *B. rotunda* rhizomes [11], *K. parviflora* rhizomes [12], *M. oleifera* leaves [13], *Z. officinale* [14], and *N. Nucifera* stamen [15]. Male reproductive abnormalities are caused by endogenous factors or exogenous toxicants [16].

Environmental toxicants and chemical exposure are contributors to male reproductive dysfunction, causing infertility [16]. MZ is widely used to control fungal pathogens in agricultural and industrial applications [17]. Despite its advantages, MZ has been found to contaminate agricultural products and the environment, leading to oxidative stress and other toxic effects in animals [18]. MZ has been reported to induce oxidative stress and cause testicular dysfunction, epididymis abnormalities, and spermatozoa abnormalities [18,19].

Vitamin E (Vit E) and vitamin C (Vit C) consumption can prevent and manage MZ toxicity [20,21]. Vit E is one of the most commonly used antioxidants used in increasing frozen sperm motility and fertility [22]. However, the natural antioxidants from plant extracts could be against chemical toxicity [14]. Thus, the natural antioxidants from plants with phenols and flavonoids might be developed and used to prevent and manage MZ toxicity. 

In Thailand, MZ is used to control fungal pathogens in agricultural and industrial applications, where people and farm animals could be subjected to continued exposure. Therefore, this study aims to investigate the phytochemical compounds and effect of white *Nelumbo nucifera* petals aqueous extraction (WNAE) on antioxidant activity, protein profile, energy preservation, and cattle sperm quality induced by MZ.

## 2. Results

### 2.1. Phytochemical Screening of WNAE Using Liquid Chromatography-Mass Spectrometry (LC-MS) 

**Figure 1:**
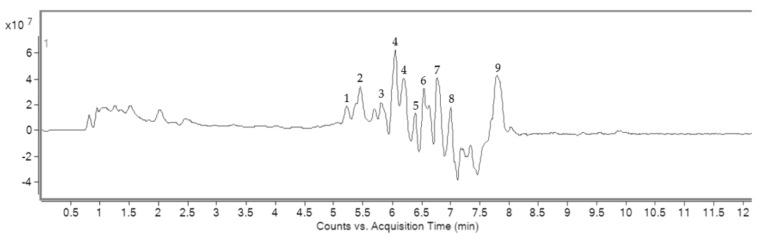
LC-MS chromatogram of WNAE showed peak identification of phytochemical compounds. Peak identification: peak 1, (+)-delta-Tocopherol; peak 2, kaempferitrin; peak 3, ouabain; peak 4, convallatoxin; peak 5, salasodine; peak 6, isorhamnetin-3-O-rutinoside; peak 7, 2′, 3, 3′ 4, 4′-pentahydroxy-4′-glucosulchalcone; peak 8, 4, 8′-Bi ((+)-epicatechin); peak 9, quercetin-3-O-arabinoglycoside.

Chromatogram showed nine phytochemical compounds, including (+)-delta-Tocopherol, kaempferitrin, ouabain, convallatoxin, salasodine, isorhamnetin-3-O-rutinoside, 2′, 3, 3′ 4, 4′-pentahydroxy-4′-glucosulchalcone, 4, 8′-Bi ((+)-epicatechin), and quercetin-3-O-arabinoglycoside (Figure 1).

### 2.2. Identification of Inhibitory Concentration 20 (IC20) 

The MTT (3-[4,5-dimethylthiazol-2-yl]-2,5 diphenyl tetrazolium bromide) assay of cattle sperm was used for the IC20 value of the WNAE, MZ, and Vit E evaluation. The mean IC20 values of WNAE, MZ, and Vit E were 0.22, 0.25, and 0.41 µg/mL, respectively.

### 2.3. Antioxidant Properties of Sperm 

**Figure 2:**
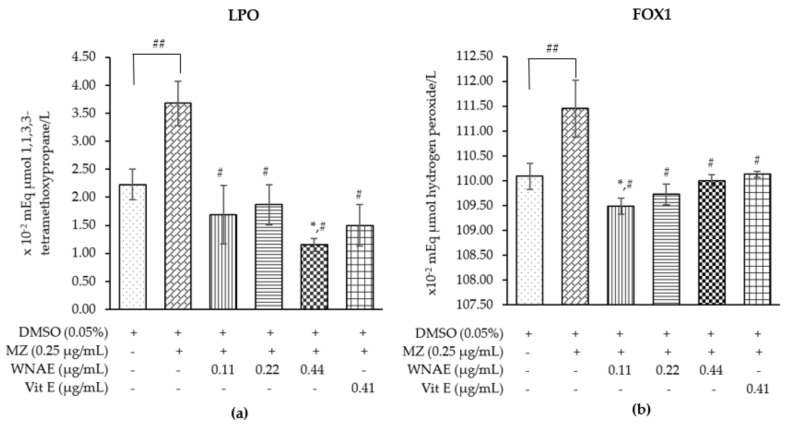
The mean value ± standard deviation (error bars) of LPO (**a**) and FOX1 or total oxidative status (**b**) of the cattle sperm sample treated as follows: control, MZ, WNAE 0.11, 0.22, 0.44 µg/mL following MZ, and Vit E 0.41 µg/mL following MZ (MZ: mancozeb, WNAE: white *N. nucifera* petals aqueous extraction). Data was obtained from three replications (*n* = 3). * Denotes the significant differences from control group at *p* ≤ 0.05. ^#^ Denotes the significant differences from MZ group at *p* ≤ 0.05. ^##^ Denotes the significant differences between MZ and control group at *p* ≤ 0.05.

The lipid peroxidation (LPO) and ferric-xylenol orange (FOX1) in cattle sperm treated with MZ, WNAE + MZ, and Vit E + MZ (positive control), and the results are presented in Figure 2.

The results of LPO concentration were shown significantly increased in the sperm treated with MZ when compared to the control group, while the sperm co-administrated with 0.11 and 0.22 µg/mL of WNAE and MZ, and co-administrated with Vit E and MZ groups were significantly lower than the MZ-treated group and similar to the level measured in the control. In addition, the co-administration of WNAE at the dose of 0.44 µg/mL was shown to be in a significantly lower concentration when compared with the MZ and the control groups but was not significantly different in the other doses and the dose co-administrated with Vit E (Figure 2a).

The effect of MZ on the FOX1 status to reflect the total oxidative status in sperm has been presented in Figure 2b. The MZ-treated group showed a significant increase when compared to the control. All doses co-administrated with WNAE and co-administrated with Vit E presented a significant decrease in the total oxidative status when compared with the control. Moreover, the dose co-administrated with WNAE at the dose of 0.11 µg/mL showed a significantly lower concentration when compared with the control group. The AOPP, AGEs, and the total antioxidant status were not significantly different in all groups. 

### 2.4. Sperm Proteins’ Profile (SDS-PAGE)

**Figure 3:**
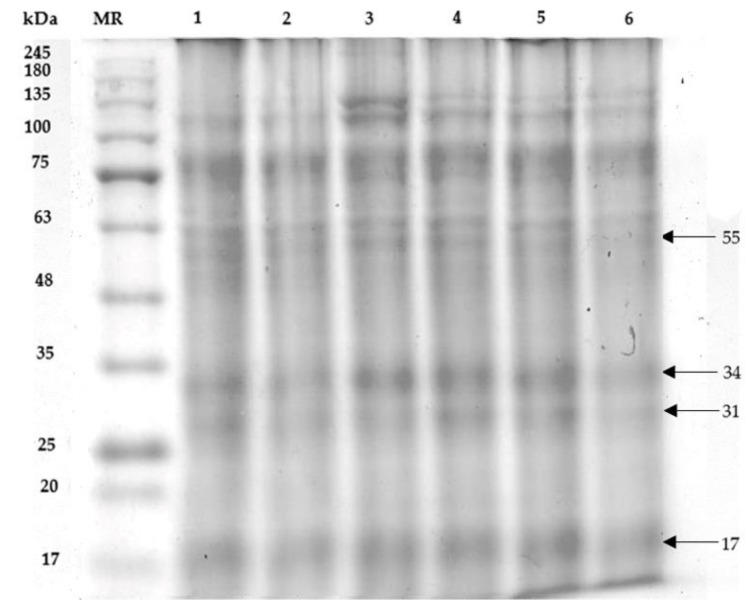
Protein profile of Charolais cattle sperm sample treated as follows: control (1); MZ (2); WNAE at the dose of 0.11 (3); 0.22 (4); 0.44 (5) µg/mL following MZ, and Vit E 0.41 µg/mL following MZ (6) (MR: protein marker, MZ: mancozeb, WNAE: white *N. nucifera* petals aqueous extraction).

The sperm proteins of Charolais cattle sperm treated with MZ and WNAE contained protein bands with various molecular weights (MWs). Four proteins were found to be associated with the sperm motility, acrosome reaction, sperm fertilization, and sperm viability had MWs with 17, 31, 34, and 55 kDa, respectively (Figure 3). The protein intensities with MWs of 17, 31, 34, and 55 kDa in the MZ-treated group showed a significant decrease when compared with control group (Table 1). All co-administrations of WNAE and the co-administrated with Vit E were significantly increased in protein intensity with MWs 55 kDa when compared to the MZ. The protein intensity with MWs 34 kDa was enhanced in all co-administrations when compared with the MZ group. All co-administrations of WNAE significantly increased in the 31 kDa of protein intensity from the MZ. Moreover, the protein intensity with 17 kDa was significantly increased in all co-administrations when compared to the MZ and the control groups.

### 2.5. Adenosine Triphosphate (ATP), Adenosine Diphosphate (ADP), and Adenosine Monophosphate (AMP) Levels

**Figure 4:**
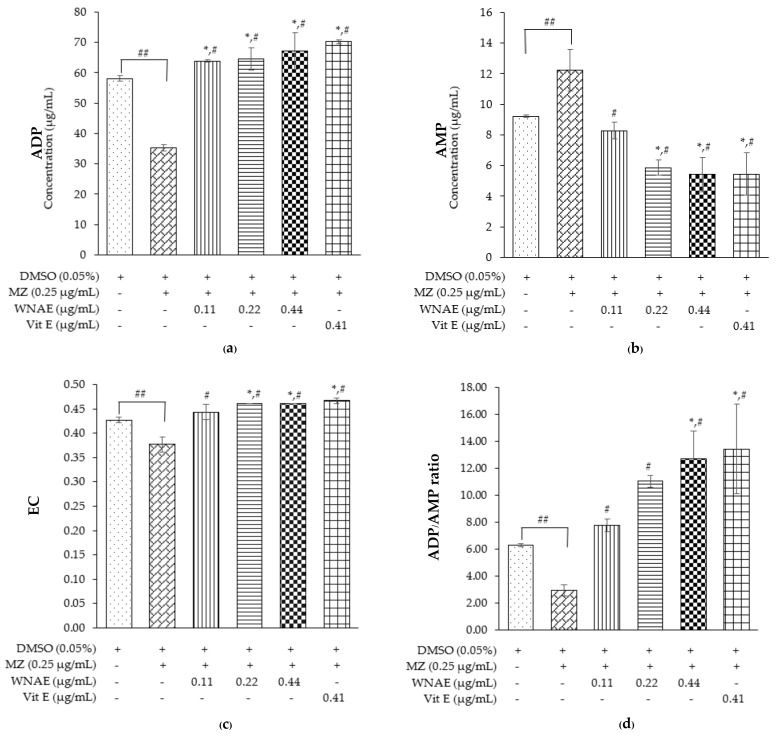
The mean value ± standard deviation (error bars) of ADP (**a**); AMP (**b**); EC (**c**), and ADP/AMP ratio (**d**). The cattle sperm sample was treated as follows: control, MZ, WNAE 0.11, 0.22, 0.44 µg/mL following MZ, and Vit E 0.41 µg/mL following MZ (MZ: mancozeb, WNAE: white *N. nucifera* petals aqueous extraction). Data was obtained from three replications (*n* = 3). * Denotes the significant differences from control group at *p* ≤ 0.05. ^#^ Denotes the significant differences from MZ group at *p* ≤ 0.05. ^##^ Denotes the significant differences between MZ and control group at *p* ≤ 0.05.

The HPLC method was applied to determine the concentrations of ATP, ADP, and AMP in the sperm sample. The ATP levels could not be detected in this cattle sperm model, thus ADP, AMP, EC, and ADP/AMP ratio was measured. The ADP levels were significantly lower in the MZ-treated group when compared with other groups, while co-administrated of WNAE and the co-administrated with Vit E were shown to be significantly higher than the control group (Figure 4a). Conversely, the results of AMP levels were shown to be significantly high in the MZ-treated group when compared with the other groups, while the co-administrated of WNAE and the co-administrated with Vit E were significantly lower than the control except the co-administrated of WNAE at the dose of 0.11 µg/mL (Figure 4b). The results of EC (Figure 4c) and the ratio of ADP/AMP (Figure 4d) showed a significant decrease in the MZ-treated group when compared to the control group. All groups of co-administrated doses of WNAE and the co-administrated with Vit E showed a significant increase when compared to the MZ-treated group. The results of EC showed a significant increase in the co-administrated of WNAE at the dose of 0.22 and 0.44 µg/mL, and co-administrated with Vit E when compared with the control groups, while the ratio of ADP/AMP showed a significant increase in the co-administrated doses of WNAE at the dose of 0.44 µg/mL and co-administrated with Vit E.

### 2.6. Sperm Quality Tests

#### 2.6.1. Sperm Motility

The value of sperm motility was presented (Table 2). Six treatments were prepared as follows: T1 = Krebs solution (control); T2 = MZ 0.25 µg/mL; T3 = WNAE 0.11 + MZ 0.25 µg/mL; T4 = WNAE 0.22 + MZ 0.25 µg/mL; T5 = WNAE 0.44 + MZ 0.25 µg/mL, and T6 = Vit E 0.41 + MZ 0.25 µg/mL. The progressive movement and non-motility of sperm was significantly decreased in the groups of sperm induced by MZ when compared to the control group. All co-administrated with WNAE and co-administrated with Vit E can decrease the non-motile sperm movement when compared with MZ group, while the dose co-administrated with WNAE at the dose of 0.11 µg/mL was significantly decreased than the control group. 

#### 2.6.2. Sperm Viability and Acrosome Integrity

**Figure 5:**
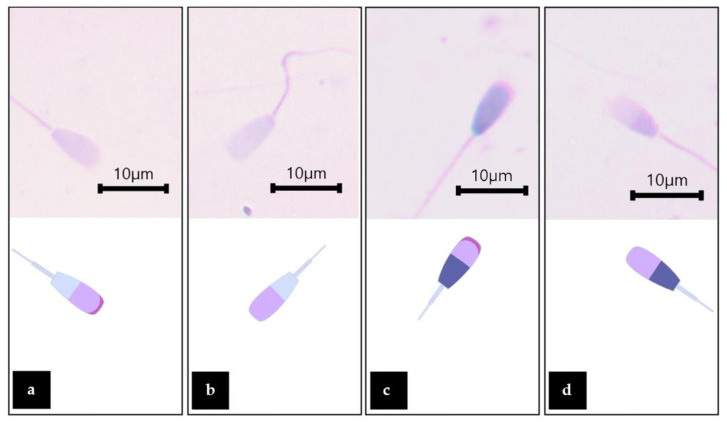
Viability and acrosome integrity classification of Charolais cattle sperm stained with TB/Giemsa: Sperm and graphic drawings were demonstrated as viable sperm with intact acrosome (**a**); Viable sperm with detached acrosome (**b**); dead sperm with intact acrosome (**c**), and dead sperm with detached acrosome (**d**) showed at a magnification of 1000×.

The analysis of the number of sperm viability and acrosome integrity in the different treatments demonstrated in Table 3. The MZ-treated group showed a significantly lower number of viable sperm with intact acrosomes when compared with the control group. However, the co-administrated dose of WNAE and the co-administrated dose with Vit E was not significantly different in the control and the MZ. All groups were not significantly different in the other parameters. The viability and acrosome integrity of Charolais cattle sperm are demonstrated in Figure 5). 

#### 2.6.3. Sperm Morphology

**Figure 6:**
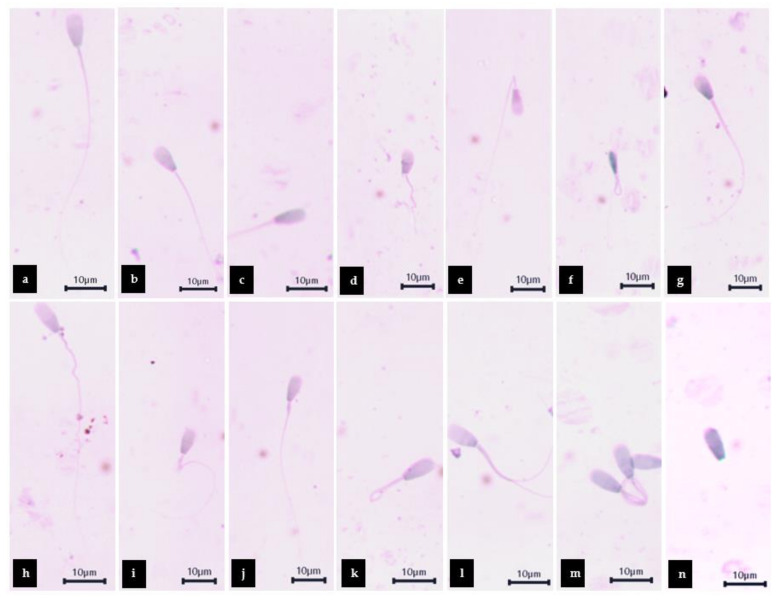
Morphological classification of Charolais cattle sperm stained with TB/Giemsa: Normal sperm (**a**); Sperm with different patterns of abnormal head (**b**,**c**): pear shaped head (**b**); Tapered head(**c**), Sperm with different patterns of abnormal head and tail (**d**–**g**): short head with irregular shaped tail (**d**); Tapered head with bent tail (**e**); Microcephalus head and hairpin tail (**f**); Macrocephalus head with edema tail (**g**), and sperm with different patterns of abnormal tail (**h**–**n**): irregular shaped tail (**h**); Bent tail (**i**); Thick midpiece (**j**); Hairpin tail (**k**); Multiple tail (**l**); Coiled tail under the head (**m**); No tail (**n**), showed at a magnification of 1000×.

Normal sperm, abnormal head, abnormal head and tail, and abnormal tail patterns of cattle sperm cells are shown in Figure 6. The number of abnormal tails was shown to be significantly increased in the MZ-treated group when compared with the control, while the co-administrated group and the co-administrated with Vit E were not significantly different in the control and the MZ-treated group. The 0.11 µg/mL co-administrated dose of WNAE showed significantly decreased in the head abnormality when compared with the control. In contrast, the 0.11 µg/mL co-administrated dose of WNAE was shown to be significantly increased in the number of normal sperm when compared with the MZ-treated group (Table 4).

## 3. Discussion

This experiment of the Charolais cattle sperm model induced by MZ was used and applied to investigate the in vitro model. The advantage of using the LC-MS method is that it can separate flavonoids and alkaloids with up to eight compounds and Vit E. Quercetin-3-O-arabinoglycoside was a major flavonoid that was found in WNAE. The previous study utilizing the HPLC method found quercetin as a major substance in the *N. nucifera* petal extract, followed by catechin, gallic acids, and *p*-hydroxybenzoic acid [5]. Kaempferitrin, isorhamnetin-3-O-rutinoside, 2′, 3, 3′ 4, 4′-pentahydroxy-4′-glucosulchalcone, 4, 8′-Bi ((+)-epicatechin), and quercetin-3-O-arabinoglycoside, which are flavonoids present in our diet and have strong natural antioxidant properties [23], have been reported to increase reproductive ability [24].

The current study found that within 3 hours of cattle sperm exposure to MZ resulted in an increase of free radicals, leading to induce LPO. Generally, lipids are the macromolecules in the living cells that form the phospholipid bilayer of the cell membrane and are the free radical targets [16]. The sperm plasma membrane contains high levels of polyunsaturated fatty acids (PUFA) that regulate membrane fluidity and permeability [6]. When the levels of free radicals are high, they attack PUFA, leading to the oxidation of lipids in the sperm membrane [16], thereby impairing the sperm membrane’s capabilities. This increased sperm damage resulting in less sperm viability [25]. Consistently, this result showed that there was decreased cattle sperm exposure with MZ viability, while the sperm induced by MZ, co-administrated of WNAE and co-administrated with Vit E, had the potential to decrease total oxidative stress and LPO levels. Similarly, the white NAE decreased LPO in the cell-free system and decreased total oxidative stress in rat sperm induced by FeSO_4_ [5]. 

From the SDS-page, the cattle sperm found four proteins related to sperm quality. There were four different protein intensities with MWs of 17, 31, 34, and 55 kDa. Proteins are the most common component in the living cells and are easy to be oxidized [7]. This study showed that protein band intensities decreased after MZ exposure and indicated that there were protein oxidations [26]. The protein with a MW of 17 kDa is called A-kinase anchor protein 3 (AKAP 3), which is located in the sperm’s tail, and regulates sperm motility [8]. The proteins with MWs in the range of 33–34 kDa are called Phospholipase C-zeta (PLCζ), which plays a role in calcium oscillation and oocyte activation that influences successful fertilization in mammals. This study found that the sperm motility and sperm fertility, and the protein intensity at an MW of 17 and 34 kDa, were decreased similar to that of the study of low-fertility in Holstein bulls [8]. 

Similar to the protein with an MW of 17 kDa, the protein with an MW of 31 kDa had the same effect on sperm. The protein with an MW of 31 kDa as a heparin binding protein, was involved in the acrosome reaction ability [9]. This experiment found that both the sperm acrosome integrity and the protein intensity at an MW of 31 kDa were reduced as the study of cold shock stress during cryopreservation of bull semen [9]. In addition, the proteins with MWs in the range of 55 kDa are related to sperm viability. Similarly, the previous study was found that the 55 kDa fraction was correlated with the sperm viability of fresh semen and predominant in higher fertility bulls [10]. However, the sperm induced by MZ, co-administrated with WNAE, and co-administrated with Vit E had the effects of reducing protein oxidation and increasing the protein intensity. Similarly, bisphenol A induced oxidative stress and loss sperm function, while glutathione and Vit E reduced oxidative stress in BPA-exposed spermatozoa [27]. Vit E and WNAE had antioxidant activity and free radical scavenging that decreased LPO and protein oxidation, leading to an increase in sperm quality. 

Sperm motility requires ATP from mitochondria [28]. The present study found that within 3 h of cattle sperm exposure to MZ, ATP was so low that it could not be detected. This evidence may be due to the use of ATP during sperm motility and its conversion to ADP by the adenylate kinase (AK) enzyme that maintains the reaction catalyzed (2ADP ↔ ATP + AMP) [29]. Thus, in this study, the ADP and AMP could be investigated, with ADP decreasing and AMP increasing in the sperm treated with MZ. Moreover, the sperm exposed to MZ reduced EC and ADP/AMP ratio because MZ was found to decrease the potassium in ion channels’ activity [30], resulting in decreased sperm motility [31]. Nevertheless, the ADP level, EC, and ADP/AMP ratio were increased in the sperm induced by MZ, co-administrated with WNAE, and co-administrated with Vit E, but the AMP level was decreased because Vit E and flavonoids in WNAE extract are strong antioxidants [20,22,23,24,32] with the potential for free radical scavenging, resulting in increased energy preservation, sperm motility, sperm viability, acrosome integrity, and normal sperm morphology. 

The sperm quality studied included sperm motility, sperm viability, acrosome integrity, and normal sperm morphology and were listed in decreasing order in the group induced by MZ within the 3-hour timeframe. These results were similar to the in vitro study for the acute effect of MZ on bull sperm for 2 h, which resulted in decreased sperm function, sperm motility, membrane integrity, and acrosome activity, but no effects for the exposure in 0.5–1 h range [33]. In addition, the male rats receiving MZ at a dose of 800 mg/mL are able to pass the effects to their offspring, such as decreased sperm motility and sperm viability [19]. Therefore, it is interesting to apply WNAE in combination with MZ in animal models. The quality of the sperm, especially acrosome integrity, plays an important role in the oocyte penetration process, and serves in the motility and the fertilization ability [26]. MZ contains heavy metals including zine and manganese, and the metabolization of MZ produces ethylenethiourea (ETU), which can generate free radicals [16]. Therefore, MZ exposure can have a negative effect on the male reproductive system in both in vitro [33] and in vivo conditions [18]. However, this study presented the groups of sperm induced by MZ co-administrated with Vit E as having had a positive effect and enhanced normal sperm morphology, sperm viability, and acrosome integrity. These effects were the same as taking Vit E, as is commonly used as a positive control, because it is an effective antioxidant for deceased oxidative stress, preventing cell damage, and increasing sperm quality in frozen sperm and azoospermia samples [10,27,28]. The present study showed the WNAE enhanced the sperm motility, normal sperm morphology, sperm viability, and acrosome integrity during the oxidative stress process, suggesting that the crude extract in WNAE contains bioactive compounds that might act with direct effect on the scavenging process of the free radicals in cattle sperm induced by MZ, which was similar to the study in rat sperm induced by ferrous sulfate heptahydrate (FeSO_4_) [5]. Therefore, the compounds of WNAE also have a benefit effect against cell damage. 

Although Vit E has the potential to scavenge free radicals and increase energy preservation and sperm quality, this potential was shown to be less effective than co-administration with WNAE due to WNAE being found with flavonoid bioactive compounds that are a group of polyphenolic compounds abundantly present in our diet. It has strong antioxidants that neutralize the energy of the radicals and protect healthy sperm, which are efficient free radical scavengers better than Vit E. The flavonoids have more capacity than Vit E because one Vit E molecule can scavenge two radicals, while flavonoids can scavenge up to twelve radicals each [23]. Moreover, alkaloids and Vit E were found in WNAE. Alkaloids were beneficial for sperm preservation [34]. Therefore, WNAE has the potential to effectively scavenge free radicals and increase sperm quality in the vitro model more than Vit E, due to the three compounds of WNAE in the form of crude extract being more effective than isolated compounds [35]. From the results, it may be suggested that the WNAE had benefits for sperm preservation and the reproductive health for animals or humans exposed to MZ contaminants. 

## 4. Materials and Methods

### 4.1. Chemicals and Reagents

Hydrogen peroxide (H_2_O_2_), Trichloroacetic acid (TCA), and glacial acetic acid were purchased from Merk KGaA (Darmstadt, Germany), and D-Sorbitol, xylenol orange, potassium hexacyano ferrate, thiobarbituric acid (TBA), quinine hemisulfate, gallic acid, and chloramine-T were obtained by purchasing from Sigma-Aldrich (St. Louis, MO, USA).

### 4.2. Plant Material and Extraction

White *N. nucifera* petals were collected in September 2019 and originated from the Thung Yang subdistrict, Laplae district, Uttaradit Province, Thailand (17°31009.7″ N, 99°59001.6″ E). A voucher specimen number 023248–2 were identified, deposited, and authenticated at Herbarium, Faculty of Pharmacy, Chiang Mai University. The aqueous extract was acquired from *N. nucifera* dried petals. The extract was dried by lyophilization with 12.5% yield and stored in –20 °C before experimentation.

### 4.3. Phytochemical Screening by LC-MS Analysis

The phytochemical compounds of WNAE were determined by semi-quantitative LC-MS analysis. The LC-MS condition comprised of an Eclipse plus column (100 × 2.10, 1.8 µm), and the column temperature was maintained at 40 °C. The mobile phase contained 5 mM ammonium format with 0.1% formic acid in water (A) and 0.1% formic acid in acetonitrile (B). The B was started at 5% and increased to 90% in 13 min with a flow rate of 0.3 mL/min, and the injection volume was 5 µL. The chemical constituents of the extract were identified by comparing the retention times and mass spectral with the data in the Mass Bank high quality mass spectral database.

### 4.4. MTT (3-[4,5-Dimethylthiazol-2-yl]-2,5 Diphenyl Tetrazolium Bromide) Assay

Frozen semen straws from a Charolais cattle were purchased from Namchuea Wongwi Company Ltd. (Bangkok, Thailand) The frozen-thawed semen was centrifuged at 1755 rpm for 5 min and sperm pellet was washed twice with Krebs solution, pH 7.4. The sperm concentration was determined and adjusted to 10 × 10^6^ sperm/mL.

The sperm solution 100 µL was added to 96 well-plates. The Krebs solution and dimethyl sulfoxide (DMSO) 0.05% were added in the control wells. Ten concentrations of MZ, WNAE, and Vit E were added to the sperm solution and incubated at 37 °C for 3 h. The concentrations were chosen based on preliminary studies. After incubation, sperm viability was determined by MTT viability assay [36]. The results were calculated for each concentration as a percentage of control and 20% inhibition concentration (IC20) values were established.

### 4.5. Experimental Design

The frozen sperm concentration was prepared to 80 ×10^6^ sperm/mL in Krebs solution. A 500 µL was added to separate test tubes. Six treatments were prepared as follows: T1 = Krebs solution (control); T2 = MZ 0.25 µg/mL; T3 = MZ 0.25 + WNAE 0.11 µg/mL; T4 = MZ 0.25 + WNAE 0.22 µg/mL; T5 = MZ 0.25 + WNAE 0.44 µg/mL, and T6 = MZ 0.25 + Vit E 0.41 µg/mL. All treatments were incubated at 37 °C for 3 h. The experiment was done in three replications. The 20 µL of mixture solution for sperm motility studies and 20 µL of mixture solution was stained with 20 µL of trypan blue for sperm viability studies. Then the solution was centrifuged at 1755 rpm for 5 min for separated supernatant and sperm pellet. The sperm pellet was washed twice with following MZ phosphate-buffered saline (PBS), pH 7.4. In addition, the supernatant and sperm pellet were stored at −20 °C until the antioxidant properties and sperm protein profiled were assessed.

### 4.6. Antioxidant Properties of Sperm Assay

#### 4.6.1. Lipid Peroxidation (LPO) Assay

In total, 100 µL supernatant of sperm in Krebs solution was assessed by the determination thiobarbituric acid-reactive species (TBARS) assay; the method followed by Laoung-on et al. [5]. After the experimental procedure, the supernatant was investigated by a microplate reader (Bio Tek Synergy H4 Hybrid Microplate Reader, BioTek Instruments, Winooski, VT, USA) at 532 nm. 

#### 4.6.2. Inhibition of Advance Oxidation Protein Products’ (AOPP) Formation

A total of 100 µL of supernatant of sperm in Krebs solution was procured and the method was done according to the procedure of Laoung-on et al. [5]. The mixture solution was measured by a microplate reader at 340 nm.

#### 4.6.3. Inhibition of Advance Glycation End Products’ (AGEs) Formation

A 96 well-plate contained 100 µL of supernatant in each well and were determined as AGEs by a microplate reader at excitation wavelength 360 nm and emission wavelength 460 nm [5]. 

#### 4.6.4. Ferric-Xylenol Orange (FOX1) Assay and Ferric Reducing Antioxidant Power (FRAP) Assay

Total oxidant status determination was assessed by the peroxide content. The FOX1 and FRAP regent and the experimentation process were performed similar to the previous study [5].

### 4.7. The Extraction and Analysis of Sperm Proteins (SDS-PAGE)

The sperm pellet suspensions were washed three times in PBS (pH 7.4) and resuspended in lysis buffer containing glycerol, β-mercaptoethanol, 0.5 M Tris [hydroxylmethyl] aminomethane buffer (pH 6.8), 10% sodium dodecyl sulfate (SDS), and 0.5% bromophenol blue solution. The mixture was boiled at 95 °C for 4 min and centrifuged at 12,000 rpm for 10 min. The supernatant was collected for sperm protein analysis by SDS-PAGE.

SDS-PAGE was performed for separation and determination of molecular weight of sperm proteins with the stacking gel containing 4% polyacrylamide and the separating gel containing 10% polyacrylamide. A total of 15 µL of protein samples were loaded into each well and subjected to electrophoresis at 90 V for 4 h. The gel was stained with 0.1% Coomassie blue and protein fractions were compared with marker proteins (BLUelf Prestained Protein Ladder, Bio-Helix, Taipei). The intensity of the band thickness was measured using ImageJ software.

### 4.8. Determination of ATP, ADP, and AMP Levels

ADP and AMP levels were determined by reversed-phase high performance liquid chromatography (HPLC)-diode array (Agilent 1260 Infinity Binary LC, Santa Clara, CA, USA) and the Purospher® Star PR-8 end-capped column (150 × 4.60, 5 µm). The mobile phase consisted of 0.1 M diammonium hydrogen phosphate (NH_4_)_2_(HPO_4_), pH 6.0, maintained for 10 min. The spectra between 200 and 400 nm were obtained, and at 220 nm and 254 nm were examined. The chromatographic peaks were identified by comparing the retention times, and the concentrations of ADP and AMP were measured using the external standard method [37]. The energy charge was calculated as follows:

EC = [ATP] + 0.5 [ADP]/[ATP] + [ADP] + [AMP]

### 4.9. Sperm Quality Tests

#### 4.9.1. Sperm Motility

The 20 µL of mixture sperm solution was collected from each test tube and then the sperm solution was dropped into an improved Neubauer hemocytometer for motility analysis at a magnification of 400× under light microscope (Olympus CH2). A total of 200 sperm cells were counted and classified per each test tube [38].

#### 4.9.2. Sperm Viability and Acrosome Integrity

To determine sperm viability and acrosome integrity, 20 µL of sperm solution was mixed with 20 µL of trypan blue (TB). The 10 µL of this mixture was placed and smeared on the slide [39]. Slides were air-dried and fixed in fixative solution (86 mL of 1N HCL, 14 mL of 37% formaldehyde solution, and 0.2 g neutral red) for 4 min. Subsequently, it was stained with 7.5% Giemsa solution at 40 °C for 4 h, then rinsed in tap and distilled water, and air-dried. The sperm viability and acrosome integrity were examined at a magnification of 1000× under light microscope. A total of 100 sperms were counted and classified per each test tube [40].

#### 4.9.3. Sperm Morphology

The slide of sperm stained with TB/Giemsa was used for morphology determination. The sperm morphology was evaluated and classified at a magnification of 1000× under light microscope. Sperm morphological classification was divided into four patterns: the normal, the sperm with abnormal head, the sperm with abnormal head and tail, and the sperm with abnormal tail [38]. A total of 100 sperms were counted and classified per each test tube [41].

### 4.10. Statistical Analysis

The data presented mean ± standard deviation (SD). Excel Microsoft 365 was used for the inhibitory concentration 20 (IC20) determination. The normal distribution was determined via Kolmogorov–Smirnov test. The statistical analysis of the mean values of AMP level, ADP/AMP ratio, LPO, viable sperm with intact, and number of normal were using one-way ANOVA followed by Tukey’s tests to analyze the differences between groups. The Kruskal–Wallis, followed by Mann–Whitney U tests were carried out to analyze the differences between groups of mean values of other parameters. The independent *t*-test or Mann–Whitney U tests were used in the comparison between MZ and control groups. SPSS 22.0 was applied for all statistical analyses. All significance levels were inferred at *p* ≤ 0.05. 

## 5. Conclusions

In conclusion, WNAE contained flavonoid bioactive compounds and had potential more than Vit E for free radical scavenging in the cattle sperm exposed to MZ. The WNAE enhanced sperm motility, sperm viability, acrosome integrity, and normal sperm morphology by scavenging free radical and increasing antioxidants. From the results, it may be suggested that the WNAE had benefits for sperm preservation. The reproductive health in animals or humans exposed to MZ contaminants should be further studied.

## Figures and Tables

**Table 1:** The proteins band intensities of Charolais cattle sperm sample treated with mancozeb (MZ), white *Nelumbo nucifera* aqueous extraction (WNAE), vitamin E (Vit E), and the control group.

Molecular Weight (kDa)	Intensity (× 10^3^ a.u.)					
	Control	MZ (0.25 µg/mL)	MZ + WNAE (0.25 + 0.11 µg/mL)	MZ + WNAE (0.25 + 0.22 µg/mL)	MZ + WNAE (0.25 + 0.44 µg/mL)	MZ + Vit E (0.25 + 0.41 µg/mL)
55	26.81 ± 0.57	11.39 ± 0.57 ^##^	26.71 ± 0.64 ^#^	22.04 ± 0.68 *^,#^	32.15 ± 0.35 *^,#^	23.89 ± 0.64 *^,#^
34	53.87 ± 0.65	37.44 ± 1.62 ^##^	76.79 ± 2.35 *^,#^	50.65 ± 0.19 *^,#^	55.26 ± 1.61 ^#^	50.3 ± 1.26 *^,#^
31	79.89 ± 0.65	49.77 ± 0.64 ^##^	73.46 ± 1.17 *^,#^	91.23 ± 1.51 *^,#^	75.81 ± 1.95 *^,#^	47.03 ± 0.38 *^,#^
17	51.87 ± 0.99	31.11 ± 0.34 ^##^	68.1 ± 0.21 *^,#^	66.83 ± 2.67 *^,#^	69.35 ± 0.54 *^,#^	72.73 ± 2.08 *^,#^

Proteins at molecular weight at 55, 34, 31, and 17 kDa were analyzed using *Kruskal–Wallis* test followed by Mann–Whitney tests at *p* ≤ 0.05. Data are mean values ± standard deviation and three replications (*n* = 3). * Denote the significant differences from control group at *p* ≤ 0.05. ^#^ Denote the significant differences from MZ group at *p* ≤ 0.05. ^##^ Denote the significant differences between MZ and control group at *p* ≤ 0.05.

**Table 2:** Number of motile and non-motile sperm of Charolais cattle sperm sample treated with mancozeb (MZ), white *Nelumbo nucifera* aqueous extraction (WNAE), vitamin E (Vit E), and the control group.

Group	Number of Motile Sperm		Number of Non-Motile Sperm
	Progressive	Non-Progressive	
Control	4.22 ± 4.87	14.33 ± 5.98	181.44 ± 7.92
MZ (0.25 µg/mL)	0.00 ± 0.00 ^##^	2.67 ± 1.22 ^##^	197.33 ± 1.22 ^##^
MZ + WNAE (0.11 µg/mL)	0.44 ± 0.73	27.11 ± 6.95 *^,#^	172.44 ± 7.33 *^,#^
MZ + WNAE (0.22 µg/mL)	0.78 ± 1.39	21.89 ± 5.93 *^,#^	177.33 ± 6.04 ^#^
MZ + WNAE (0.44 µg/mL)	0.33 ± 0.50	24.33 ± 4.30 *^,#^	175.33 ± 4.15 ^#^
MZ + Vit E (0.41 µg/mL)	0.33 ± 0.50	16.89 ± 2.80 ^#^	182.78 ± 2.91 ^#^

Number of motile and non-motile sperm were analyzed by Kruskal–Wallis test followed by Mann–Whitney U test. Data was obtained from nine replications (*n* = 9). * Denotes the significant differences from control group at *p* ≤ 0.05. ^#^ Denotes the significant differences from MZ group at *p* ≤ 0.05. ^##^ Denotes the significant differences between MZ and control group at *p* ≤ 0.05.

**Table 3:** Numbers of sperm viability (viable and dead) and acrosome integrity (intact and detached acrosome) of Charolais cattle sperm sample treated with mancozeb (MZ), white *Nelumbo nucifera* aqueous extraction (WNAE), vitamin E (Vit E), and the control group.

Group	Number of Viable Sperm		Number of Dead Sperm	
	Intact	Detached	Intact	Detached
Control	40.78 ± 15.28	14.00 ± 9.44	32.89 ± 17.81	12.22 ± 7.28
MZ (0.25 µg/mL)	13.44 ± 7.29 ^##^	11.22 ± 14.32	58.67 ± 22.53	16.56 ± 10.41
MZ + WNAE (0.11 µg/mL)	29.55 ± 17.63	8.44 ± 5.96	49.22 ± 23.37	12.78 ± 5.63
MZ + WNAE (0.22 µg/mL)	34.44 ± 16.52	10.00 ± 8.28	41.89 ± 21.93	12.56 ± 11.44
MZ + WNAE (0.44 µg/mL)	28.78 ± 19.43	12.11 ± 8.43	48.11 ± 28.27	10.89 ± 7.15
MZ + Vit E (0.41 µg/mL)	26.13 ± 19.63	7.25 ± 3.33	50.87 ± 16.04	16.00 ± 8.52

Numbers of sperm viability were analyzed by one-way ANOVA followed by Tukey’s test. Data were obtained from. nine replications (*n* = 9). ^##^ Denotes the significant differences between MZ and control group at *p* ≤ 0.05.

**Table 4:** Numbers of the normal and abnormal sperm of Charolais cattle sperm sample treated with mancozeb (MZ), white *Nelumbo nucifera* aqueous extraction (WNAE), vitamin E (Vit E), and the control group.

Group	Number of Normal Sperm	Number of Abnormal Sperm		
		Head Only	Head and Tail	Tail Only
Control	77.67 ± 5.75	1.78 ± 0.83	3.78 ± 6.53	17.11 ± 7.99
MZ (0.25 µg/mL)	72.00 ± 5.94	1.77 ± 1.86	1.55 ± 1.59	24.88 ± 4.31 ^##^
MZ + WNAE (0.11 µg/mL)	83.78 ± 4.99 ^#^	0.33 ± 0.71 *	0.44 ± 0.53	15.44 ± 5.15
MZ + WNAE (0.22 µg/mL)	76.33 ± 8.00	0.89 ± 1.05	1.11 ± 1.05	21.67 ± 6.75
MZ + WNAE (0.44 µg/mL)	74.67 ± 9.77	1.78 ± 1.72	1.00 ± 1.12	22.56 ± 9.10
MZ + Vit E (0.41 µg/mL)	73.38 ± 5.93	3.25 ± 6.02	0.88 ± 1.13	22.50 ± 9.84

Number of normal sperm analyzed by One-way ANOVA followed by Tukey’s test; number of abnormal sperm were analyzed using Kruskal-Wallis test followed by Mann-Whitney U test). Data were obtained from. nine replications (*n* = 9). * DenotesDenote the significant differences from control group at *p* ≤ 0.05. ^#^ DenotesDenote the significant differences from MZ group at *p* ≤ 0.05. ^##^ DenotesDenote the significant differences between MZ and control group *p* ≤ 0.05.

## Data Availability

The authors declare that the data supporting the findings of this study are available within the article.
